# Migrant Parents of Adolescents With School Refusal: A Qualitative Study of Parental Distress and Cultural Barriers in Access to Care

**DOI:** 10.3389/fpsyt.2019.00942

**Published:** 2020-01-09

**Authors:** Lucie Rosenthal, Marie Rose Moro, Laelia Benoit

**Affiliations:** ^1^ Department of Child and Adolescent Psychiatry, Toulouse University Hospital (CHU de Toulouse), Toulouse, France; ^2^ Department of Child and Adolescent Psychiatry, Centre Hospitalier Spécialisé Pierre-Jamet, Fondation Bon-Sauveur d’Alby, Albi, France; ^3^ Maison des Adolescents - Maison de Solenn, Hôpital Cochin, APHP, Paris, France; ^4^ Dept of Child and Adolescent Psychiatry, University of Paris, PCPP, Boulogne-Billancourt, France; ^5^ Center for Research in Epidemiology and Population Health (CESP), Paris-Sud and UVSQ Medical Schools, French National Institute of Health and Medical Research (Inserm), Villejuif, France

**Keywords:** school refusal, parents’perception, adolescents, transcultural, cross-cultural, migrant families, misdiagnosis, access to care

## Abstract

**Introduction:** School refusal is an important problem in adolescent psychiatry. However, little is known about the experience of school refusal among minority youth (migrants and minority ethnic groups). This study assesses how parents of various cultural backgrounds experience their adolescents’ school refusal.

**Method:** This qualitative study is based on interviews of 11 parents of teenagers diagnosed with school refusal at three adolescent outpatient mental health units in Paris and its suburbs. Interpretative phenomenological analysis was used for the thematic investigation.

**Results:** The analysis found four themes: (i) confronting school and school refusal distresses parental representations; (ii) school refusal as a failure of the family’s obligation to succeed after migration; (iii) representations of school that fluctuate with time since arrival: idealization, followed by mistrust and disappointment in the inequalities, even the racism; (iv) solutions envisioned for school refusal, confronting the healthcare system, stigma, and, again, inequality.

**Conclusion:** All parents question their parenting choices when their children become school refusers. However, when families belong to minority groups, school refusal calls into question parents’ relations with the French school system and their immigration choices. At the same time, the construction of a multicultural identity for children and adolescents in transcultural situations requires them to strike a balance between two worlds, and school refusal endangers this delicate negotiation. Subsequent misunderstandings can lead clinicians to misdiagnose school refusal as truancy. Clinicians must take the parents’ culture and migration history into account to minimize the risk of complete failure of treatment for school refusal and the ensuing inequality of care and opportunity that can result.

## Introduction

School refusal affects around 1% of pupils and accounts for 5% of the children seeking psychiatric consultation (1). Berg’s consensual (2) definition of school refusal includes (a) reluctance or refusal to attend school, often leading to prolonged absences, by children who (b) stay home during school hours with their parents’ knowledge rather than concealing the problem from them, (c) experience emotional distress at the prospect of attending school (somatic complaints, anxiety, and unhappiness), (d) do not show severe antisocial behavior, and (e) whose parents have made reasonable efforts (parental pressure) to secure the child’s attendance at school (3, 4).

Adolescence is a sensitive period during which the risks of serious consequences of school refusal are highest (5–7). Without adequate treatment, most youths with school refusal continue to have school attendance problems and emotional distress (8), leading to short- and long-term adverse outcomes such as anxiety disorders (9), depression (10), unemployment, socialization disorders, and a higher risk of developing a psychiatric disorder (11–13). Early intervention is required, and prognosis depends on how much school the child misses (14). School refusal both affects and involves the adolescents’ families, and family characteristics are one of the four categories of risk factors for school refusal; the others are the individual, the school, and the community (15, 16).

Historically, family functioning has been considered a central cause of school refusal (14, 17). Researchers focused first on the preponderance of phobic disorders among mothers of girls with school refusal (18), subsequently arguing that parental disorders, family structures, and relationships play a determinative role in the frequency or severity of school refusal: parental lack of self-esteem (19), psychiatric disorders such as anxiety, depression, or stress (16), and marital disagreement or parental inconsistency (20). Nonetheless, some school refusal cases have not appeared to involve any family pathology or dysfunction and show the need for a broader understanding of these issues (5).

Societal changes affect not only adolescents, but also their parents, and the relationships between them. Expectations about the roles of parents continue to expand; they are today supposed to be good teachers as well as good parents (21). Some aspects of modern life favor school refusal, including but not limited to the prolongation of compulsory schooling and increasing school competition (22). French public schools, which were designed to—and did for a long time—function as a social ladder, are now criticized as an outdated system that mostly reproduces inequalities (23). Although school refusal spares no socioeconomic environment, it is clearly those from lower socioeconomic levels who are in need of guaranteed equal access to care.

Cultural variables play a role in school refusal. Yamada highlighted the role of the school model in current Japanese culture and the pressures, uniformity, and extreme competition it induces. He also pointed out marked societal changes: growing urbanization, a rising divorce rate, the spread of the nuclear family model, and the parallel decline of the traditional extended family structure (24). Kenji Kameguchi, considering school refusal to be a revolution in this Confucian society, has proposed family treatment of school refusal that focuses not on the child but on strengthening the parents’ relationship (25).

Some cultural categories of students are overrepresented in a topic that is closely related to school refusal but different: absenteeism. In the United States, national absenteeism statistics (for students aged 9–10 and 13–14 years) find rates of 25%–29% among Native Americans, 21%–24% among Hispanics and African-Americans, 18%–19% among whites, but only 12%–13% among Asians and Pacific Islanders (26). School absenteeism principally concerns children coming from poor families, leading some authors to point out the economic and cultural dimensions of not completing school (27, 28). These dimensions apply to most aspects of school success. Among the 15–16-year olds in the French public school system, 25% have an immigrant background; at the same time, the French school system capacity for integration is mediocre at best (29). In France, these young people not only encounter academic difficulties, but also have a higher risk of grade repetition and dropout ideation than native-born students (30).

In 2008, Christopher Kearney stressed that the “cross-cultural aspects of … school refusal behavior remain in need of greater exploration and explication” (31). Since then, only a few papers have raised the question of culture and school refusal. According to Benoit, the school refusal of migrant adolescents might reveal their fear of losing their family cultural codes through academic and social achievement (32). Marie-Rose Moro’s team at the *Maison des Adolescents* (an outpatient unit dedicated to psychiatric care of adolescents) has been working for years with migrant adolescents and children, as well as with the children of migrants (33). Preadolescence and adolescence are especially vulnerable periods for children with this background (34). Moro links this vulnerability to the gap experienced by migrant children between their “inside world, linked to the affectivity and cultural universe of parents” and their “outside world, of school and media” governed by the norms of the host country (35). For children of migrants, schooling also means becoming part of French institutions, learning their language and their codes (36).

During adolescence, as part of the process of identity construction, teens come to question their vision of their parents as strong and reliable. In migrant families, this questioning may be increased because the culture codes of the parents are not relayed by the host country (37). Adolescent empowerment and peer group affiliations cause these teens to call into question these two worlds and the balance between them; migrants’ adolescent children must negotiate their identities carefully (38, 39). The not infrequent weakening of these families by events causing migration can impede this negotiation and leave the adolescents caught in a conflict of loyalty between these two worlds, in which the school may take the symbolic place of the *outside world,* the host country. These cross-cultural issues can lead to various negative school events, such as failing examinations or school refusal (40).

Few studies on school refusal have focused on parents who are immigrants to the country they live in, and none specifically on parents’ experiences. Parents are distressed by their children’s inability to go to school, their fear of school. This situation calls their parenting ability into question, at a minimum in their own minds. For a youth with school refusal, parents are the primary interlocutors of both school and clinicians. Thus, cultural differences cannot be ignored in the parents’ dialogue with health care, social work, and educational institutions.

This study explores the experience of migrant parents of adolescents diagnosed with school refusal. It identifies the common aspects of these experiences, the meanings that these parents attribute to their children’s school refusal, and their pathway to care. Improving our understanding of migrant parents’ behavior towards their teens and health care institutions will help to provide equal and early access to care for every child with school refusal.

## Materials and Methods

This qualitative, phenomenological, and inductive study explores parental representations of school refusal in a transcultural context. The central role of empirical results combined with an inductive process enables original findings (41). A phenomenological framework, because of its similarity to the approach used in clinical psychology and psychiatry, appears most appropriate for studying the experience of distress (42). The choice of this methodology allows us to explore here the subjective experiences of parents in all their diversity, to approach the sensitive issue of their cultural origins, and to understand the relation between these origins and their experience of their children’s school issues.

### Sampling

The research group initially recruited young people with school refusal; their parents were secondarily recruited for this study. The adolescents were aged 12 to 21 years old, had been diagnosed with school refusal according to Berg’s criteria (detailed in the first paragraph of the introduction) and receiving psychiatric care for more than 6 months. They had multicultural origins, defined as having at least one parent from a different culture, that is, different from that of metropolitan France. French overseas departments and territories were considered to be different cultures. This definition was deliberately broad. Sampling was purposive, and the subjects selected were representative of typical cases (43). The contact was made *via* the psychiatrists seeing each youth. They presented the research project, provided the youth with an information sheet for themselves and their parents, and then asked their young patients to participate. The adolescents were separately interviewed for another study by the research group. The youths were recruited from three different *Maisons des Adolescents* (adolescent outpatient psychiatric units) (33): Maison de Solenn (Hospital Cochin, Paris), Casita (Hospital Avicenne, Bobigny), and Casado (Hospital St Denis, St Denis), the latter two in disadvantaged inner suburbs northeast of Paris.

### Data Collection

Once the adolescent agreed to parental interviews, research team members called the parents to provide information about the study and set up a meeting for face-to-face semistructured interviews of around an hour. Each parent was interviewed alone at the hospital, in a public place (cafe), or at their home. The interviews were conducted from January 2017 through April 2018, by one of four different people: a child psychiatrist (LB), and three residents working on different topics of the same research project (among them LR). The interview guide was designed by the research group for this study (**Table 1**). During the interview, the researcher explored the parents’ narratives of their children’s history of school refusal, their representations of the schools involved, the school system, and their children, and their relationships with the French public institutions providing education and health services. All interviews were audio-recorded and transcribed verbatim, in full.

**Table 1:** Interview guide (starter questions).

1. Current family situation - How old is your child? Does he/she have brothers and sisters? What is your marital and professional situation? Currently, how are things going for him/her?
2. History of the disease, understanding of the disorder - When did your child start being afraid of school? Were you the only one worrying? Did someone draw your attention to his/her difficulties? Do you remember what worried you first? What drew your attention to the problem? When and how did you understand his/her difficulties? How did your spouse (his/her mom or dad) understand them? Your family?
3. Relationships with school - How did the school react to your child’s school refusal? Were you able to talk to the teachers? The school nurses? The school doctors?
4. Relationships with care - When did you decide to consult a health professional? Which one? Did you get any advice? What kind of care was provided?
5. Cultural specificities - How many languages does your child speak? When did you decide to come to France? How was the journey? What does your family think about your child’s fear of school?

### Ethical Standards

This study was carried out in accordance with the recommendations of an appropriate ethics review board (Inserm ethics review board, IRB 00003888) with audio-recorded consent. All subjects (adolescents and their parents) gave informed consent for the research and for the publication of the datasets (characteristics of the study population and direct quotations from the participants) in accordance with the Declaration of Helsinki and French law. All interviews have been anonymized and the datasets deidentified.

### Analysis

The analysis applied the method of interpretative phenomenological analysis (IPA), which enables the study of the meaning subjects construct from their experiences (44). Meticulous analysis of the interviews as each was completed enabled us to identify a set of superordinate themes, each linked to several themes describing all of the experiences narrated (45). Factors considered in selecting the themes included but were not limited to the frequency with which they were mentioned, the richness of the passages illustrating them, and how they illuminated other aspects of the narrative.

The principal researcher, LR, analyzed the interviews to optimize the validity of the results. The interviews were independently coded by two researchers (LR and LB). Codes were discussed during group meetings to enable triangulation, which both enriches the analysis and serves as a quality control process (46). The methodological criteria were retrospectively verified according to the COREQ (Consolidated criteria for Reporting Qualitative research) checklist.

## Results

### Population

Eleven migrant parents of adolescents with school refusal completed interviews. Their characteristics are summarized in **Table 2**. The teenagers easily agreed to participate and let us question their parents; but it was more difficult to include parents.

**Table 2:** Characteristics of the study population.

N	Sex	Children first name	Place of birth	Cultural particularity	Spouse place of birth	Spoken language at home	Recruitment area
P1	F	Merlin	France		Albania	French	75
P2	F	Dalla	Mali	Soninke	Unknown	French	93
P3	F	Leila	Algeria	Kabyle	Algeria	Kabyle	93
P4	F	Lea	France		Italy	French	75
P5	F	Ana	Spain		Portugal	French	75
P6	M		Portugal		Spain		
P7	F	Michael	France		Martinique	French	93
P8	M		France	Martinique	France		
P9	F	Akash	Sri Lanka	Tamil	Sri Lanka	Tamil	93
P10	M		Sri Lanka	Tamil			
P11	F	Abdel	Algeria		Morocco	French and Arab	93

This table summarizes the social and demographic characteristics of the study population.

Three couples were interviewed, separately: the parents of Ana, Michael, and Akash. Ana’s parents both arrived in France as children. Her mother (P5) was born in Spain, her father (P6) in Portugal. Michael’s parents are both French nationals. His mother (P7) was born in metropolitan France, of modest Breton origin. His father (P8) was born in Martinique, as were both paternal grandparents (of African and Indian origin). Akash’s parents are both Tamils from Sri Lanka and arrived in France in the 1990s as adults. Only the father speaks a few words of French; the mother does not speak French at all. Each was interviewed with an interpreter.

In the remaining families, only mothers could be interviewed. Abdel’s mother (P11) was born in Algeria but describes herself as half-Moroccan, half-Algerian. His father was born in Morocco but has Algerian nationality. Merlin’s mother (P1) is French, and his father Albanian. Leila’s mother (P3) is Algerian of Kabyle origin. She refused to let us question her husband, also Kabyle. Lea’s mother (P4) is French and her father Italian. Lea refused to let us interview her father because of family tensions.

The parents were separated in only one family. Dalla’s mother (P2) was born in Dakar, Senegal, and belongs to the Soninke community. Dalla’s father, with whom Dalla has almost no contact, comes from the same community, as does her stepfather.

### Thematic Analysis

The phenomenological thematic analysis of the interviews enabled us to uncover 10 themes, organized around four superordinate themes. The first shows how school refusal disrupts parental representations; the second that families perceive school refusal as their failure to achieve their obligation to succeed, to improve their children’s future and opportunities by their migration; the third concerns the fluctuations of parents’ representations of French schools between idealization, mistrust, and disappointment; and the last brings together the various solutions, standard, unusual, or traditional, that parents considered and their representations of them; these include the discovery of adolescent psychiatry, confrontation with otherness, the difficulty of access to care, and exploration of other types of care including “traditional” methods.

#### The Disruption of Parental Representations by School Refusal

##### Culture as a Strong Element of Identity

For some parents, the culture of the country of origin is a source of comfort; for others, these memories are painful, marked by war, violence, or poverty. Some of them fled war, with no choice. The country’s historical or current problems remains a source of anxiety in some parents’ discourse, even after migration, especially when relatives still live there.

Certain character traits, described as specific to the culture of the birth country, have a strong identity value. Parental attitudes may thus be legitimized by cultural affiliations. One mother explained that the distance between her husband and her children and the gendered distribution of parental duties are due to his southern Italian origins.

*He’s having a hard time finding his place in all this…. He is a typical Italian dad; *the* mother raises the kids while the dad works (P4).*

Religious faith can be a way of nurturing a cultural attachment and sense of belonging to the country of origin. Some parents, however, feel that their religious practices are irreconcilable with the standards of the host country. This raises questions about cultural transmission and strategies of integration.

##### Cultural Transmission and Integration Strategies

While some parents take on their cultural affiliations as facets of their identity, others teach their children that it is better to keep them private, at home. They may present risks for the child in the host country: these transmitted practices must paradoxically be both preserved and concealed. Thus, Leila’s mother teaches her daughter how to live her religious beliefs or traditions covertly, separating her private life practices from what she does in the outside world.

*I’ve told her many times, “My darling, if you want to apply your religion, go to Algeria! There we apply it, it’s halal, it’s the headscarf, you dress as you want, but here if you want to succeed in your life you have to blend in!” (P3).*

Another strategy parents teach their children is to cultivate a judicious distance towards the cultural logic, especially that of the country of origin. One mother, for example, described the caste system of her Soninke ethnic group with a critical tone and a sense of complicity with the interviewer, while underlining her membership in a superior caste. Children who have grown up in a different country can be critical of its traditional systems.

##### Cultural Confrontation Calls the Parents’ Cultural Logic Into Question

The family roles that they are expected to play in the host country can be new for the parents. Childcare professionals in schools and in health care facilities remind parents of these differences and of the role they are supposed to adopt in the host country. School refusal challenges what it means to be a child, to be a parent, and to become an adult. It causes parents to question their family organization and upsets them, for they do not know how to be the kind of parents they feel expected to be in France. The birth country solutions to school refusal are often inapplicable. Alongside this other school culture emerges another definition of parenthood.

Adhering to the norms of parenthood of their birth country might even break the law in this different place. Parents may feel completely paralyzed in their parental role, unable even to advise their child.

*At home, if we didn’t go to class, the parents would hit us, tie us up, they would beat us saying: “You have to go to class”. But here, if we beat him, we’ll end up in jail…. So, I cannot do anything, I cannot ask him, I cannot talk to him, because he gets angry and … I cannot even try to start a discussion, or push him a little … (P10).*

Their children’s grasp of some norms and not others confuses the parents. Some young people surprisingly claim their parents’ cultural models. One adolescent explained to his mother that stopping school would not be a problem, because he would embrace Albanian culture, living in the same house as his parents. She reported what he said to her:

*Albanian women stay with their parents … I’ll go get an Albanian woman and then I’ll stay with you (P1).*

##### School Refusal and Culture Refusal

Sometimes, school refusal accompanies the adolescent’s lack of interest in the customs of their family culture. This rejection can be very painful for parents with a strong cultural identity. When it happens, they always express their hope that it is a temporary phenomenon.

Some adolescents even demonstrate hostility toward any cultural affiliations with the country of origin. The Tamil parents developed few cultural connections to their new country, barely speaking French. They relied on their son to mediate everything. He did it all, but he refused to show any interest in Tamil customs or culture:

*He’s not interested in the Tamil world, he doesn’t watch movies, music. Even when there’s a party, he sits, he doesn’t move (P10).*

#### School Refusal as Parental Failure to Meet the Obligation to Succeed

##### High Expectations, Special Children Who Carry the Old Country Within and Must Succeed in the New Country

Nearly all of these parents had great hopes for these idealized children with bright futures, and all the children had been worthy until school refusal occurred. They were the pride of their parents, admired by family, friends, and teachers. In this exile, some children carried their parents’ dreams of a better life. The Tamil mother admitted:

*I imagined he could study engineering, before, I dreamed that he could go for advanced schooling, that he … I put all my dreams, in fact,… in him (P9).*

The children often made these requirements of excellence their own; most demanded high academic results of themselves. Many parents pointed out that the demands for success did not come from them but from the child. Some children themselves had special ambitions, aspiring to attain a higher socioeconomic level than their parents and qualify for prestigious occupations: architect, engineer, airline plane, or upper level jobs in the luxury industry.

Some parents explained the special status of their child by their belonging to a significant caste in the country of origin. At a minimum, children are supposed to maintain the social status they had in the old country. Dalla’s mother explained that her daughter belongs to the leading caste:

*She’s part of the caste … Dalla, she’s a princess, in fact, in her father’s village, and him, he is … king, these are the leaders of the village (P2).*

When these very high cultural expectations are combined with a surface strategy of hyper-adaptation to the host country, parental demands appear paradoxical— to be proud and hide at the same time. This can lead to resistance by adolescents, who may choose to assert their family cultural identity and blame their parents for not doing so. This can cause real conflict. Leila’s mother (P3) described one such argument, when her daughter was in the hospital:

*[Leila] said to me, “Why didn’t you tell them that I eat halal!” [I said], “Imposing on the hospital to make you a special [Halal] meal: are you crazy? (outraged tone) You don’t want to eat, you say ‘I’m not eating.”… Sometimes she tells me “you’re not a good Muslim.”*

Even if they are not the oldest, these youths have had the status of an oldest child, recognized for exceptional qualities that have earned them their parents’ trust. More than their siblings, they have been able to understand both the inner and the outside world. For the families where the parents do not speak French, the oldest has indeed been a translator and a guide for the new country.

*Since the beginning, since the day he could read, since then, he’s always helped the family to do everything…. All the questions at the bank, he explains everything. Yes, he looks at the accounts, he says, “Here is, there’s the problem.” He has managed everything since always (P9).*

He did not share his siblings’ childish behavior, but behaved as an adult who has and meets his responsibilities, to the point of parentification, that is, reversing the roles of the generations.

The families who immigrated earlier, where the parents themselves were educated in France, had more moderate expectations of their children. These parents, having endured great pressure for academic success from their own parents, have been careful not to impose this on their children.

Some parents perceive the adolescent’s academic failure as a major blow, a hurt commensurate with the hopes and dreams they had for him or her. The children change from over-invested, supported in all their projects, to … nothing. Many parents pointed out the strangeness of this failure for a child who has the ability to succeed brilliantly at school. The parents, family, teachers, and family doctors share the shock and disappointment of the demotion, as does the teenager. As Michael’s mother said:

*He had tremendous potential, because he has enormous potential, but hey, that’s it, it’s not … basically it didn’t work out (P8).*

The child’s failure feels to the parents like their own, aborting the social elevation they sought for the next generation. The culmination, the goal of the difficult journey of migration was supposed to be their children’s success.

##### The Parent’s Perceived Obligation to Their Social Circle and Their Family to Succeed

The expectations of academic success are thus part of a broader obligation of social success; children are called upon to climb the social ladder, but above all, they must not lose their initial social status. It is unthinkable for the parents to have a status in the host country inferior, as judged by those in the old country, to what they had had there.

Among the families that immigrated early, the parents we interviewed were themselves educated in France on their arrival. In these families, different representations of school coexist. The parents feel out of step with their family: their own parents, the grandparents of these adolescents, lived at a time when, if they even could go to school rather than work, severe corporal punishment was common, and parents always considered the teacher to be right. The more recent immigrants may even have experienced this more severe schooling in their birth country. Ana’s mother describes her parent’s school:

*When the student misbehaved, [the teacher] put him in a corner that had salt on the floor and made him kneel on it. It was very hard to stay in that position…. Parents listened to the teachers, the teachers were right and they determined your future (P6).*

From the perspective of these grandparents (and newer immigrants), going to school is an opportunity and the problem of school refusal appears completely incomprehensible, especially at a school with no corporal punishment. This intergenerational gap in school representations is found in both the French-educated families participating in the study and among families of newer migrants. This mother explains how the Italian grandmother does not understand her granddaughter’s school refusal:

*She didn’t study at all, she went to school very little, and her life was rough, complicated. And I think she would like everything to work out, but she doesn’t understand all that (P4).*

This was true for all the grandparents mentioned in interviews.

The parents’ representations of school are different from those of both their parents and their children. This gap leads to such opposition that a three-generation dialogue on educational and academic problems is difficult, if not impossible. Parents made it clear that their own parents are not a source of further understanding about the problem of school refusal, to whom they could turn for help. Many have thus abandoned the idea of making their parents understand their children’s difficulties. The corollary of this silence is the loneliness of both the parents and grandparents, isolated from one another. Dialogue between them ends, for silence is preferable to conflict. To protect themselves, the parents say nothing or even lie. The family validation of their own parental role is at stake and endangered by the children’s school refusal. This father clearly explains that abandoning his son’s schooling means dishonoring the whole family, especially himself.

*No, I didn’t talk about it, because otherwise … Even the high school diploma, some people asked:” did he get it? We said yes because otherwise it’s a big deal. We don’t talk about it…. He has to succeed so that there’s pride. People won’t say that he is sad, that he thinks about this or that or I don’t know what. They’ll just say that the parents don’t know how to raise their child … It’s very hard for me … It’s as if, the fact that my son does not succeed, it’s as if I had failed something, especially the oldest child (P10).*

The failure of his eldest son puts his own obligation to succeed at stake. His own family laid this obligation on him when he migrated; he cannot fail. The only way for this father to avoid stigma is to lie.

#### Representations of School: Idealization, Mistrust, and Disappointment

##### Disappointment

Some parents, because they immigrated as adults, or because they lack an academic education, knew nothing about the French school system and idealized it. In general, parents counted on the school to enable their children to climb the social ladder.

Most were aware of the inequalities in schools and their success (or failure) rates. Often dissatisfied with the school maps, they looked for the best possible institution. They sought the best possible conditions for their child: tranquility but also access to a certain level of shared culture.

Some parents had learned that French schools were far from ideal during their own childhood, when they arrived at an early age and began school. Several fathers and one mother had faced discrimination in schools that had oriented them early on towards vocational classes and hurt them deeply all along the way. The racist clichés and discriminatory actions of teachers and other pupils intensified language problems. Ana’s father endured jokes about the Portuguese; Abdel’s mother could not find a single apprenticeship in hairdressing in Lille.

*Every time I went for an internship … I was told “We don’t take North Africans.” It disgusted me and I gave up (P 11).*

Many viewed the school system as racist, implicitly ranking immigrants according to context and country of origin. They were wary of the school environment, where discrimination is implicit if it is not explicit. It seemed inevitable to most of the parents interviewed that their children would someday be victims of racism.

*The fact that he is mixed-race, precisely, I paid special attention to that because I know it’s a delicate matter (P7).*

This mother, married to a native of Martinique, then implied that it is easier to be of Korean origin than of African and Martiniquais origin, that is, that being Asian is generally better accepted than being black. Another mother explained that her daughter has her father’s Portuguese last name and is thus entitled to several nicknames and repeated jokes about Portuguese *concierges* (caretakers).

The higher the level, the more discriminatory schools become. Michael began his school refusal in architecture school; his mother explained that these schools are less diverse; she worried about the trivialization of racist clichés that her son innocently reports to her.

##### Know the School and Its Limits, Adapt, and Act

Communication with the school requires several prerequisites, which the schools themselves do not appear to be aware of. The need to decode what goes on and is said in school is obvious to families who have been living in the country for a long time, especially when the parents attended school here. The situation is quite different for families who have migrated only recently, especially if they come from a country with a radically different school culture.

The overall school institution is culturally coded; being able to identify the different interlocutors requires specific knowledge of its organization. The example of the Tamil family is especially striking because it is the only family in which neither parent speaks fluent French. They were summoned to be informed of their son’s severe difficulties by a school psychologist using an interpreter, by telephone. They did not understand the psychologist’s function and had never seen her before. These communication difficulties resulted in shocking and sudden awareness of their son’s problems. They understood the extent of these problems and how long they had been present only later; they felt completely shunted aside by the school. Indeed, we only understood what happened because a Tamil interpreter was present during the research interview. The father received text messages that his son was absent but lacked the linguistic and cultural knowledge to perceive the implicit content of the messages.

One criterion of knowledge of the system is the ability to challenge it. Being able to look critically at the school system and find the best solution for one’s children requires extensive knowledge. For example, Ana’s mother had a friend who was a Spanish teacher, who criticized the private school that both their children attended, specifically on the basis of a teacher’s inadequate written French. To be able to observe these errors requires, quite obviously, excellent mastery of the French language. The mother, Spanish and long well-integrated, has no illusions about the integrating role of this school. It is clear to her that the school will not understand or care about cultural differences; she knows it’s impossible:

*The teachers are overwhelmed … It’s a Catholic school, but there are Muslims, there are Jews, there’s a little bit of everything (P5).*

Some informed parents choose private schools, as Ana’s father did, to protect their children from the French public-school system, the racism of which he had experienced and suffered from when he was a child:

*We decided to put them in a private school because it would be better for them, they would have normal lessons, they would be in a good school, a good class, with good pupils. So that they would have what I did not (P6).*

#### Solutions Envisioned for School Refusal and Their Representations

##### Encountering the Mental Health System: Calling Otherness Into Question Again

Lacking the necessary institutional codes makes a relationship to mental health care similar to that to the school. Parents sit at consultations in a discreet, withdrawn posture for fear of making a mistake or breaking some taboo and being judged a bad parent. Professionals sometimes misinterpret this attitude and think that the parents are uninvested, although it is actually a sign of respect and an effort not to interfere. Hospitals and clinics, like schools, are public establishments, a symbol considered equivalent to French culture.

The rules of the health care system can unintentionally violate various cultural representations of parenting. The concept of majority that gives young people access to medical confidentiality can distress a Tamil woman as a mother by excluding her from her child’s care. Some parents felt stigmatized because of their cultural difference and perceived a need to defend themselves. Families are often struck by the systematic questions about their own childhood or their way of life when they do not, at least at first, see that these have any association with their children’s difficulties. Their cultural difference is thus perceived as a possible flaw in their parenting. Stigma can extend to factors besides cultural origin, such as family models. A mother stigmatized because she grew up in foster or residential care has similar difficulties in defending herself from an insistent psychiatrist or social worker.

##### Real Inequalities in Access to Health Care

Standing up to judgments and to cultural stigma requires self-assurance on the part of parents. Some families question the relevance of care, but questioning is an ability that must be acquired; as for the school system, knowledge is necessary. School refusal often seems treatment-resistant, and the parents’ helplessness is reflected by the failure of psychiatry in their discourse.

Knowledge of the health care system is an evident advantage in obtaining quick access to specialized structures. A strong relational network makes it possible to find an appropriate facility. Ana’s parents have been in France since they were children and have an extended social network in Paris. A friend recommended the *Maison des Adolescents*, and they obtained an appointment within a reasonable delay.

By contrast, the lack of both knowledge and a network resulted in a very long delay in appropriate care for the Tamil family, isolated and unable to communicate easily in French. Their child Akash was not referred to the *Maison des Adolescents* until very late, after almost two years of severe absenteeism, on the eve of his baccalaureate examination. This delay was due mainly to communication problems with the school.

##### Exploring Other Solutions Outside the Standard French System

Traditional or “old-country” therapies appeared to be a taboo subject. While Leila talked easily about the different explanations of her difficulties by her family in Algeria and the possibility that someone cast a hex on her, her mother was defensive about the idea, responding vaguely, vacillating between agreement and refusal. The mother quickly dropped this hypothesis, attributing it to her own mother or husband and positioning herself outside these cultural representations:

*That’s what my mom told me … my husband agrees with you. He says it’s not their fault. It’s like you say, it’s not on purpose. But after all, I don’t know what to think (P3).*

Leila’s mother was uncomfortable talking about traditional Kabyle practices, but we know from the interview with Leila that a traditional ritual was performed to protect her. The mother expressed a pejorative view of those popular beliefs: “if we listened to them,” “them” as opposed to herself, “a believer” with a strong Muslim identity. She contrasted Kabyle rituals to Muslim belief and minimized their impact.

Abdel’s mother also quickly refuted the evil-eye hypothesis. She made fun of it, labeling it “that rigmarole.”

*Yes, I must admit, when my son wasn’t feeling well after the wedding, I thought about it. I thought about it. After, it’s over. I thought maybe at the wedding, there were many family members and all that. It’s true with us, there is a lot of rigmarole. I thought about it (P11).*

Akash’s father also avoided the question of traditional therapies for his son. He imagined a somatic cause for his son’s school refusal. This representation was invalidated by the French model, where anxiety is represented as something psychological instead.

*Me, I proposed to go see a specialist, a neurologist, and I was told that it was useless, that it was not the problem, but that it was rather here, that it was work that’s not so neurological or … medical at this level, but rather psychiatric (P10).*

Akash’s mother was much more at ease describing the care they sought. Less defensive, she spontaneously explained that they saw a traditional Tamil priest who was both an astrologer and numerologist. His divination linked Akash’s problems to the position of the stars, unfavorable to him. She described the prescriptions she followed in both countries, for the priest recommended prayers in France and offerings in Sri Lanka.

Nonetheless, the hypothesis of a vulnerability due to migration emerges from her discourse, linking the fatigue that prevents her son from going to school to a difficulty in reconciling two poles, hot and cold, India and France. This problem developed when he was little. The etiological hypothesis is thus that a violent cultural blending made Akash vulnerable—the violent differences between the two countries, the two climates.

*We went to India when he was little and at that time it was very, very hot there, he ate lots and lots of ice cream, because it was very hot…. And when he came back here, he caught something. I think it’s linked to the heat and the cold here … he was hospitalized more than two weeks, he had more than 40°C fever, we had to immerse him in cold water so that his body … It was very hard for me…. But we never knew what it was. And after, it was better, but the fatigue remained, the physical fatigue (P9).*

Other families looked for new solutions inspired by other models. Some joined community groups against bullying, while others served as parents’ representatives. Those parents, extremely invested in their child’s schooling and trying to make up for the insufficient protection of the school system by intervening more at the school level, were the most socially integrated parents in the study.

For Ana’s family, which is also very well integrated, one solution considered was sending her abroad to avoid the French school system. This family knows a lot about other existing school systems. They claim that their migratory experience is a strength that will allow Ana to travel if she wants and find another solution, elsewhere, if no solution in the French system suits her. The father has traveled repeatedly back and forth between France and Portugal, claiming this freedom with a certain pride.

*If that’s what she wants and if it’s better elsewhere, yes of course without hesitation, she’ll leave. Maybe she’ll come back, we don’t know, maybe she’ll stay or she’ll go wherever she wants. In the family we are travelers, you know. (Laughter) We’ve gone to the four corners of the world so that’s it, we are travelers (P6).*

## Discussion

### School Refusal Calls Family Roles Into Question

Mothers responded to our research with enthusiasm. Most of them gladly shared their experiences, and many pointed out at the end of the interview that they had confided more than they had thought. They transmit their birth country culture and those speaking a different language teach it to their children: Spanish for Ana, Tamil for Akash, Arabic for Leila and Abdel. They also transmit coping and adaptive strategies to their children to help them avoid discrimination. These strategies reveal their representations of the French system; because they think the system is intolerant of their Muslim religion, both Abdel’s and Leila’s mothers have taught their children to remain discreet and hide their differences from the outside world.

Fathers are significantly underrepresented in these interviews, perhaps reflecting a weakened paternal function. Only one family, Dalla’s, included a stepparent; all the other sets of parents were still living together. We were unable to interview half the fathers, although we repeatedly asked to do so. Lea, in conflict with her father, refused to allow us to interview him. Another father could not be reached. An appointment with Leila’s father was set up, but his wife forbade the contact. Three fathers were interviewed. Two had immigrated as children and speak excellent French; both experienced racism as children, and both were particularly concerned about their teenagers’ academic difficulties. The third had arrived more recently and spoke very little French. He actually came to the interview due to a misunderstanding: he thought he had an appointment with his son’s care team, rather than a research interview.

These interviews shed light on the fathers’ roles. In patriarchal societies, the father is the interface permitting family and society to interact (47). Migration weakens the paternal function because he lacks knowledge about the host country culture and rules; often at the same time, however, he is the breadwinner, whose work determines the family’s economic and social status. Linguistic obstacles and economic difficulties combine to call his place into question. An educational model in the host country radically different from that in the birth country can be a real challenge for paternal function. This is the case for Akash’s father who tends to withdraw respectfully. He misunderstood the apparent attitude of the French educational system, misperceiving it to prohibit him from raising his child in his own way; this withdrawal in turn led the school to view him as an uncaring or neglectful father. The original devaluation of the culture goes hand in hand with depreciation of the parental image and can result in adolescents’ absence of interest in their parents’ culture, as it did for Akash. This depreciation of the image with which the adolescent naturally identifies may be considered one key to school refusal in migrant families; it leads to a depressive affect among these adolescents, as well as to their parentification (48).

The generation of grandparents and the extended family are also concerned by school refusal. Grandparents, whether remaining in the birth country or living in the new country, mostly did not have the opportunity to attend school but worked from a very early age. They do not understand the adolescents’ problems. This misunderstanding can lead to a breakdown in relationships between grandchildren and grandparents, but also between the grandparents and their own children. Family ties are challenged by school refusal, often notably loosened. Parents sometimes even lie to their parents to hide the school problems.

Parental loss of authority sometimes results in the teenager’s parentification. The parents in this study mainly described their children by terms such as mature and responsible. Studious and trustworthy, their parents delegated many responsibilities to them. The cases of Leila and Akash are particularly representative. Leila was treated as the oldest when she is in fact the second oldest sibling, and Akash was considered to be the only child able to translate the French world to his parents and to fully understand them. He therefore became an essential interpreter of the French world for his parents. In extreme cases, this mechanism results in a paradoxical filiation (49), where the son finds himself above his father’s law. We can wonder if these adolescents’ roles as mediators, interpreters, and translators of the institutions of France might perhaps confer too much power on them, bestow an omnipotence that hinders their adaptation to the school and social systems.

### Transcultural Interpretation of Anxiety-Based School Refusal

All the adolescents involved in this study were good, even excellent students, until anxiety exploded—anxiety about school, about knowledge—and froze everything. School refusal raises questions about relations to knowledge, desire to learn, and hunger for autonomy. In the words of Margaret Mahler, migration can be thought of as a process of separation-individuation: in separating from the country of origin, the migrant also separates from internal objects related to it (50). For these second-generation migrant youth, the construction of adolescence, the desire for autonomy, the investment of the outside world, might well conflict with their loyalty to their family, to their parents’ culture. Unless they can negotiate a resolution by blending these two cultures, this conflict can substantially inhibit their cognitive and social functioning in the school environment.

All of these teenagers were enrolled in general (i.e., academic) education programs and had good grades; each had intended to continue postsecondary studies after high school. When children’s anxiety prevents them from attending middle or high school, their future—their occupation, their income, their social status—is jeopardized. Several of their parents repeatedly insisted to us on these adolescents’ wonderful qualities, describing them as brilliant youths with bright futures in professions far above those of their parents. These teens may perceive their parents to be asking their child to invest, succeed in the host society, where the parents have not achieved as much as they would have liked. This imposed obligation may be especially painful in adolescence, when the construction of identity imposes so many choices of culture, acceptance, and loyalty (37).

From a transcultural point of view, school refusal can be interpreted as resulting from the problems adolescents have in managing simultaneously their relations with their family and with the outside world of school, as well as the relations between their family and school. This difficulty is associated with a fragile representation of self, parents, and family. Another manifestation of this conflict is an oscillation between moments of rejecting their parents’ culture and returning to it. These contradictory movements accompanying school refusal upset parents. They worry that their child will no longer participate in community festivities; inversely they are surprised to see their teenager again interested in things related to the parents’ birth country. Ana’s father is surprised that she is learning Portuguese, his native language, while refusing to attend school. Leila’s case is an extreme example, with a massive cultural division separating the inside world, the house, where family members can live according to their culture and their religion, and the outside world, where they must hide any differences to blend in with the majority culture. There is no room for negotiation in Leila’s construction of identity; she must hide her profound cultural identity from the outside world. No blending or mixing of cultures appears possible. School refusal might thus be understood as a refusal of the outside world, preventing exchanges between it and her inner world and thus temporarily avoiding anxiety-inducing encounters. Extra-familial mutism can accomplish the same goal (35).

The transgenerational obligation to succeed, in the words of Lebovici (51), concerns both parents and children. It is very hard for parents to surrender their dreams for their child’s success, especially when it was often part of their reason for migration. Some parents persist in their aspirations for success although their child has refused to attend school for several months. This inability to adapt their expectations borders on denial. In other families, the failure to meet the transgenerational obligation to succeed occurred in the previous generation, when the parents, who arrived as children, disappointed their own parents’ hopes. In these families, there is less denial, more understanding, and more patience.

In France, many immigrants come from formerly colonized countries, and many more French citizens are their children or grandchildren. Four of the families in this study, that is, half the research population (or rather at least one parent from each of these families) comes from former French colonies: Senegal, Morocco, Algeria, and Martinique. Its colonial past remains a delicate topic in France. When the parents come from a former colony, however, conscious and unconscious elements of colonial history may persist in their relations with French institutions. Parental discourse may reflect this relation of domination between the host country and their birth country, for example, when it praises the host country at the expense of the birth country (52) or when the parent deliberately remains withdrawn from a conversation. This asymmetry affects adolescents’ relations with the only French institution they have to deal with: school. No parent spontaneously broached the issue of colonial history during the research interviews. It nonetheless implicitly appeared in the mothers’ repeated recommendations to their children to be polite, respectful, and discreet, to avoid any problems. These injunctions of invisibility are implicit, but omnipresent, formulated as parental expectations, advice, and strategies to cope with cultural differences.

This discretion also extended to the content of the research interviews, where parents rarely discussed their birth culture spontaneously. Only the Tamil mother mentioned on her own initiative traditional rituals that families can perform to cure the anxieties of adolescents. In his study of Algerian immigration, Abdelmalek Sayad has theorized that this phenomenon is a domination relationship typical of colonialism and extends to the French mainland territory. He denounces politeness as an instrument of exclusion from political life, a ban on taking part in public life (53). The mothers in the two families of Algerian and Moroccan origin made clear that they have enjoined their children to be discreet. Malika Mansouri links the educational difficulties of migrant children with transgenerational trauma (54). Integrating both colonial and postcolonial history in education as well as in aspects to be thought about in health care may be a key point in considering the school problems of migrant children from former colonies.

In this postcolonial study perspective, school refusal might sometimes be considered to be the resurfacing of repressed colonial memory and a way of refusing the cultural asymmetry demanded by the institution and integrated into the parental discourse.

### Limitations and Prospects

The first limitation of this study is that it cannot show the evolution of these parental representations, which will be reshaped over time. It would accordingly be interesting to repeat the interviews with the participating parents later on.

A second limitation is the small number of parents involved in transcultural situations, compared with the total number of adolescents included in the research group. Several adolescents in transcultural families refused to consent to their parents’ participation in the study. This refusal can be interpreted as an illustration of the dual process by which these adolescents identify with and in some sense reject their migrant parents. These results cannot be considered to be final or even stable as we did not attain data saturation, but several similarities in the data do allow us to sketch three ideal types of parents according to the time since they arrived in France, as we did at the beginning of the results section, in describing the population. Although including more participants would have increased the accuracy and validity of this study, this is the first examination of this topic and it appears likely that migrant parents are particularly hard to reach, given their difficulties in having their child’s anxiety-based school refusal detected/diagnosed (and the school system’s predisposition to treat the attendance problems of immigrants’ children as truancy) and in accessing mental health care and a school support system. Given these issues, 11 parents is a more than decent number of inclusions for a prospective qualitative study.

A third limitation is the lack of detailed information about these families’ socioeconomic status as well as the evident reciprocal confounding between socioeconomic and migrant status. As pointed out above, socioeconomic status is associated with school attendance and probably school refusal (26–28), and migrant status quite often associated with socioeconomic deprivation. We do know that the socioeconomic status of these families ranges from low to medium and that two of the three recruitment centers are in disadvantaged neighborhoods. Nonetheless, given that our aim was to focus on the transcultural aspects of school refusal, more socioeconomic data might only have further blurred the interpretation of our results.

### Implications for Practice: the Need to Take Otherness Into Account

Their children’s schooling is a major issue for migrant parents. While a child’s school refusal may finally mandate communication between the parents and the school, numerous obstacles remain. For many, there is the language barrier, which affected only one family in our sample but appeared nearly insurmountable. The failure to provide an interpreter for more than a year caused substantial delay in healthcare support for Akash and may have prejudiced his prognosis. This difficulty in surmounting the language barrier for any care probably explains why this family is the only non-French-speaking family we were able to recruit.

School-related cultural codes are another obstacle; texted absence messages are a prime example. When a student misses class, parents receive a text message from the school, reporting the absence. This is an implicit invitation from the institution: the school expects parents to be concerned enough to contact them and ask for an appointment. If they do not, the parents will be considered uninvolved in the child’s schooling, uninvested, and negligent if not neglectful (55). However, this discreet posture may, on the contrary, be a sign of deep respect for the educational institution by some parents of a different culture, as for Akash’s family. Bernard Lahire’s portraits of families—all of them either working-class or disadvantaged—with children failing in French schools showed that dealing with schools (that is, institutions) necessitates knowledge of the school culture to be able to decrypt its requests and meet its requirements (56). Migrant families from a distant culture and families with disadvantaged social backgrounds have analogous difficulties in understanding this culture and its codes. Families from countries with a similar school culture, such as Spain, Portugal, or Italy, do not face this problem, for their school cultures are close enough to allow their codes to be transposed to the French system. In multicultural couples, the French spouse decodes the institution if necessary. The closer the family’s school culture is to that of France, the faster school refusal is identified and dealt with.

This observation suggests that it might be possible to set up and disseminate cross-cultural mechanisms for school mediation, either individual or collective. To our knowledge, only one such program exists, METISCO; it also includes a parenting support component and training support for school professionals (36).

It is essential to take into account the cultural and identity issues involved in adolescents’ school refusal, at the individual, family, and collective levels. Psychiatrists must investigate the cultural representations surrounding school refusal of adolescents and their parents, as well as the family’s cultural representations of school. Other early intervention programs, such as for psychosis, have already been improved by including a transcultural dimension to their clinical assessment tools (57) and institutional practices (58).

Acknowledging the family’s experiences of racism and discrimination while listening to them describe these events is also compulsory. It appears to be an essential step in the therapeutic management of adolescents’ school refusal and in dialogue with their parents. Although school and health care systems are claimed to be increasingly inclusive, difference continues to incite discrimination in school (50) and in access to care. The more systematic, institutionalized use of interpreter-mediators might be useful in reducing some of these issues.

All the adolescents in our study were born in France, but the parents differ quite notably: some left their country of origin when they were children themselves to settle in France with their migrant parents, while others arrived later, already adults, without their family. The complexity of this landscape of migrant parents is a result in itself, demonstrating that short cuts and simplifications are inadequate. The first group faced language barriers as children but are now good French speakers. The question of their cultural identity only resurfaces with their children’s adolescence and school difficulties. More recently arrived families, especially from non-European cultures quite distant from French culture, lack knowledge of French codes in nearly every field, including culture, school, parenting, and society. This ignorance significantly impedes the provision of and access to care for their children. The difficulties are paradigmatic of those faced by migrant parents of a child with a mental illness. While their questions about their parenting ability vary, all of them have some. School refusal causes them to interrogate their relationship to their culture of origin, their representations, and their family system. It also systematically disrupts the family balance.

As mentioned above, we observed three different family profiles, according to their degree of familiarity with the French system. Akash’s parents arrived from Sri Lanka as adults, knowing neither social codes nor local cultural codes of France. They have no social resources outside the Tamil community and speak very little if any French. For them, migration was accompanied by idealization of the host country. They had high expectations of their child and of both the schools and health care institutions, accompanied by almost blind trust in both their son’s success and the institutions they were dealing with.

The families of Dalla, Abdel, and Leila were more familiar with French cultural codes, but they were nonetheless not their own. These three families come from non-European cultures as well as Morocco, Algeria, and Senegal. They also had high expectations of their children and school, but they were distrustful of French institutions, which had already disappointed them. They have social resources in their country of origin as well as relationships they built in the host country.

Finally, the families of Ana, Lea, Merlin, and Michael were much more familiar with the French system and its institutions. Each of these sets of parents is bicultural. Three mothers are from metropolitan France, and the others all from Europe: Spain, Italy, Albania, and Portugal. The expectations of these families are more moderate, both in regard to their children’s academic achievements and the school’s ability to educate and protect their children. These four families have well-developed French social networks and the knowledge that enables them to have a critical view of institutions.

## Conclusion

School refusal distresses parents. Our research, focused on the experiences of migrant parents dealing with the school refusal of their children, was also an invitation to think about cultural differences in providing mental health care to adolescents and in meeting with their parents.

During their narratives of this experience, these parents told us they had had to think about who their child really is and what they really think about parenting and parenthood. They also learned how to address the school and to look for solutions for school refusal. At each of these stages, their cultural representations have been called into question. This study illustrates the need to take into consideration the parents’ culture and migration history in the management of school refusal. With their children no longer able to attend school, the parents had to rethink their family patterns and parental choices, their relations to French educational institution, the histories of their families and communities. From a transcultural point of view, school refusal can be seen as the adolescent’s inability to manage their two worlds—their family and their school, at a time when identity construction requires them to negotiate between them. It can therefore be seen as a genuine failure of cultural blending.

Children’s school refusal calls into question the success of the parents’ migration, casting a negative valence on it afterwards and leaving the parents to wonder about their family patterns, their parenting choices, their relations with France’s educational and health care institutions and beyond—for school is a gateway to France itself.

## Data Availability Statement

The datasets generated for this study are available on request to the corresponding author.

## Ethics Statement

This study was carried out in accordance with the recommendations of an appropriate ethics review board (Inserm ethics review board, IRB 00003888) with audio-recorded consent. All subjects (adolescents and their parents) gave informed consent for the research and for the publication of the datasets (characteristics of the study population and direct quotations from the participants) in accordance with the Declaration of Helsinki. All interviews have been anonymized and the datasets de-identified.

## Author Contributions

The study was designed by LB, who also asked the competent institutional review board (Comité d’éthique de l’Inserm) to approve this study. The principal researcher, LR, analyzed the interviews to optimize the validity of the results. The interviews were independently coded by two researchers (LR and LB). Codes were discussed during group meetings [LR, LB and MM) to enable triangulation, which both enriches the analysis and serves as a quality control process.

## Funding

This study received funding from the French National Institute of Health and Medical Research (Inserm) through Laelia Benoit’s PhD grant and from private contributors through the Thellie Foundation for the project “Understanding pathways to care in school refusal and improving them.”

## Conflict of Interest

The authors declare that the research was conducted in the absence of any commercial or financial relationships that could be construed as a potential conflict of interest.
